# Multimorbidity and Quality of Life: The Mediating Role of ADL, IADL, Loneliness, and Depressive Symptoms

**DOI:** 10.1093/geroni/igad047

**Published:** 2023-06-04

**Authors:** Stefan Sieber, Angélique Roquet, Charikleia Lampraki, Daniela S Jopp

**Affiliations:** LIVES Centre, Swiss Centre of Expertise in Life Course Research, University of Lausanne, Lausanne, Switzerland; Institute of Psychology, University of Lausanne, Lausanne, Switzerland; LIVES Centre, Swiss Centre of Expertise in Life Course Research, University of Lausanne, Lausanne, Switzerland; Institute of Psychology, University of Lausanne, Lausanne, Switzerland; LIVES Centre, Swiss Centre of Expertise in Life Course Research, University of Lausanne, Lausanne, Switzerland; Institute of Psychology, University of Lausanne, Lausanne, Switzerland

**Keywords:** CASP-12, Longitudinal, Mediation, SHARE

## Abstract

**Background and Objectives:**

The ubiquity of multimorbidity makes it crucial to examine the intermediary factors linking it with quality of life (QoL). The objective was to examine to what extent the association between multimorbidity and QoL was mediated by functional and emotional/mental health and how these mediation pathways differed by sociodemographic factors (age, gender, education, and financial strain).

**Research Design and Methods:**

Data from Waves 4 to 8 of 36,908 individuals from the Survey of Health, Aging, and Retirement in Europe (SHARE) were included. Multimorbidity (exposure) was defined as having 2 or more chronic conditions. Mediators included limitations with (instrumental) activities of daily living (ADL and IADL), loneliness, and depressive symptoms. QoL (outcome) was assessed with the CASP-12 scale. Longitudinal model-based causal mediation analyses were performed to decompose the total association between multimorbidity and QoL into direct and indirect effects. Moderated mediation analyses tested for differences in mediation pathways by sociodemographic factors.

**Results:**

Multimorbidity was significantly associated with lower QoL (direct effect: *b* = −0.66). This association was mediated by ADL limitations (percentage mediated 0.97%), IADL limitations (3.24%), and depressive symptoms (16.70%), but not by loneliness. The mediation pathways were moderated by age, education, financial strain, and gender.

**Discussion and Implications:**

ADL, IADL, and depressive symptoms are crucial intermediary factors between multimorbidity and QoL in older European adults, with changing importance according to age, education, financial strain, and gender. The findings may help to increase the QoL of individuals with multimorbidity and redirect care efforts to these factors.

**Translational Significance:** Understanding which factors may help to maintain or increase the quality of life (QoL) of individuals with multimorbidity is essential to improve their successful aging perspectives. Findings show that ADL, IADL, and depressive symptoms mediated the association between multimorbidity and QoL by 20.9%, with depressive symptoms being the most important (16.7%). This may contribute to the design of future care programs, making depressive symptoms a priority when aiming at the improvement of QoL in individuals with multimorbidity. Moreover, the mediation patterns varied across age categories, level of educational attainment, financial household strain, and gender, showing the importance of considering sociodemographic factors.

## Background and Objectives

Against the backdrop of a continuously shifting age structure in European societies toward growing numbers of older people, research in multimorbidity has been gaining interest (Navickas et al., 2016). With the increasing population age, the share of individuals living with multiple diseases has been documented to rise, particularly from the age of 50 years (Hopman et al., 2016; Salive, 2013; Souza et al., 2021). Population estimates indicate that about 50 million people in Europe live with multimorbidity (Rijken et al., 2016). Multimorbidity is understood as the presence of multiple chronic diseases and is commonly defined as the occurrence of two or more chronic diseases within the same person (Demirer et al., 2021; Makovski et al., 2019; Navickas et al., 2016; Rijken et al., 2016).

At the societal level, multimorbidity represents an important issue of interest because increasing rates have resulted in rising health care expenditure (Demirer et al., 2021; Rijken et al., 2016). At the individual level, multimorbidity is of high importance due to its effects on quality of life (QoL), as complex health conditions lead to increased symptom burden with deleterious effects (Demirer et al., 2021; Hopman et al., 2016; Makovski et al., 2019; Navickas et al., 2016; Rijken et al., 2016). A meta-analytic study has found that QoL decreased between 1.55% and 4.02% with each added disease an individual declared, depending on the QoL measure analyzed (Makovski et al., 2019). Given the high prevalence and ubiquity of multimorbidity in European societies, it is crucial to understand the mechanisms linking multimorbidity with QoL. Better grasping which factors may help to maintain or increase the QoL of individuals living with multimorbidity is essential to improve their successful aging perspectives.

The objective of the present study was to examine the association between multimorbidity and QoL among adults aged 50 years and older as well as to investigate to what extent this association was explained by various relevant mediators. As there is little research investigating the multimorbidity–QoL link and potential mediators, and longitudinal studies examining these associations are especially scarce (Makovski et al., 2019), we used a large international and longitudinal dataset to test a complex set of models, as shown in Supplementary Figure 1.

Among the potentially modifiable mediators that are on the causal pathway between multimorbidity and QoL are functional health factors such as activities of daily living (ADL) and instrumental activities of daily living (IADL) as well as emotional/mental health factors including loneliness and depressive symptoms (Supplementary Figure 1B). Multimorbidity may lead to limitations in ADL and IADL due to increased symptom burden hindering individuals living with multiple diseases to continue a self-sustaining way of life (Hopman et al., 2016; Makovski et al., 2020; Navickas et al., 2016), which in turn may negatively affect QoL (Navickas et al., 2016). For instance, research has shown that patients with multimorbidity had worse ADL and IADL compared to patients without multimorbidity (Williams & Egede, 2016). Based on this literature, we hypothesized that ADL and IADL both mediate the association between multimorbidity and QoL independently from each other and the other mediators.

Similarly, research has shown that multimorbidity is linked to emotional and mental health factors, with individuals having multimorbidity reporting more loneliness and depressive symptoms (Demirer et al., 2021; Hopman et al., 2016; Read et al., 2017; Stickley & Koyanagi, 2018). Concomitantly, loneliness and depressive symptoms have been shown to negatively affect QoL (Beridze et al., 2020; Demirer et al., 2021; Sivertsen et al., 2015). Although loneliness has frequently been shown to be associated with depression, loneliness is a distinct construct of depressive disorders and is also commonly experienced by older adults without depressive disorders (Donovan & Blazer, 2020). Thus, we hypothesized that loneliness and depressive symptoms both mediate the association between multimorbidity and QoL independently from each other and the other mediators.

Furthermore, apart from illness-related factors on the pathway between multimorbidity and QoL, the needs of people with multimorbidity may also depend on sociodemographic characteristics (Hopman et al., 2016; Wagner et al., 2022). Therefore, we were interested in investigating whether the mediated link between multimorbidity and QoL differed depending on age, gender, education, and household financial strain (Supplementary Figure 1C). We hypothesized that with increasing age the mediation pathways would become more important, particularly through functional health (ADL/IADL), due to the accumulated associated burden, and given that age-associated loss of compensatory resources (e.g., cognitive and social), captured by chronological age as a proxy, may increase the effect of multimorbidity on QoL (Jopp & Rott, 2006; Smith et al., 2002). Similarly, we hypothesized that gender would affect the mediation pathways, with stronger pathways for women, since the effect of multimorbidity on QoL has previously been found to be stronger among women (Makovski et al., 2019). The prevalence of multimorbidity tends to be lower among higher educational levels (Makovski et al., 2020; Pathirana & Jackson, 2018). Thus, we hypothesized that the mediation pathways would become more important with increasing educational levels, as individuals with higher education live less commonly with multimorbidity and may therefore experience a stronger impact on QoL due to their relatively healthier peers. Finally, given that the prevalence of multimorbidity has been found to be higher in lower-income households (Makovski et al., 2019, 2020; Wagner et al., 2022) and the potential additional financial burden caused by treatment and loss of work (Larkin et al., 2021), we hypothesized that household financial strain would also affect the mediation pathways between multimorbidity and QoL. More specifically, we expected that the mediation pathways may be more important for households with higher financial strain, particularly through depressive symptoms, because the increased financial burden may amplify worries related to it.

## Research Design and Methods

Data were drawn from the Survey of Health, Aging, and Retirement in Europe (SHARE), a longitudinal and population-based study on noninstitutionalized adults aged 50 years or older living in 27 European countries and Israel (Börsch-Supan et al., 2013). SHARE has collected 8 waves of data between 2004 and 2020 (every 2 years) using computer-assisted personal interviewing in participants’ homes, including sociodemographic and health-related information. The survey was approved by the relevant research ethics committees in the participating countries, and all participants provided written informed consent. Data from Waves 4 through 8 were used, as information on the mediator loneliness was collected from Wave 5 and a temporal lag between predictor, mediator, and outcome was introduced (Supplementary Figure 2). To be included in the present study, participants needed to have provided at least one assessment of multimorbidity (exposure) at one study wave (*W*), as well as ADL, IADL, loneliness, and depressive symptoms (mediators) at a subsequent study wave (*W* + 1), and QoL (outcome) at two waves later (*W* + 2) across three consecutive waves (Supplementary Figure 2). A total of 61,739 observations from 36,908 individuals (56.6% women) across 14 countries (Austria, Belgium, Czech Republic, Denmark, Estonia, France, Germany, Israel, Italy, Luxembourg, Slovenia, Spain, Sweden, and Switzerland) were included in the final sample.

### Exposure: Multimorbidity

Chronic conditions were assessed through the question: “Has a doctor ever told you that you had or do you currently have any of the conditions [listed] on this card?” Sixteen chronic conditions were included in the multimorbidity measure (refer to Supplementary Material for the full list and more details). Multimorbidity was defined as the coexistence of two or more chronic conditions (Demirer et al., 2021; Makovski et al., 2019; Navickas et al., 2016; Rijken et al., 2016), and was assessed in Waves 4, 5, and 6 (Supplementary Figure 2).

### Mediators

All mediators were measured in Waves 5, 6, and 7 (Supplementary Figure 2).

The ADL index assesses limitations with six everyday self-care activities which are fundamental for maintaining independence, including dressing (including putting shoes on), walking across a room, bathing or showering, eating (such as cutting up your food), getting in or out of bed, and using the toilet (including getting up and down), for which participants indicated whether they can perform them independently (yes = 0; no = 1; Taylor et al., 2007). The index was included as a continuous variable in the analyses ranging from 0 to 6.

The IADL index assesses limitations with instrumental activities of everyday life, considering nine activities for which respondents indicated whether they were able to perform them independently (yes = 0; no = 1): Preparing a hot meal, doing personal laundry, doing work around the house or garden, shopping for groceries, making telephone calls, taking medications, managing money (such as paying bills and keeping track of expenses), leaving the house independently and accessing transportation services, and using a map to figure out how to get around in an unfamiliar place (Taylor et al., 2007). Doing laundry and use of transportation were assessed from Wave 6 on. The index was included as a continuous variable in the analyses ranging from 0 to 9.

For Loneliness, the three-item loneliness scale was used (Hughes et al., 2004), which is a short version of the revised UCLA loneliness scale (Russell et al., 1980). The scale is comprised of three items answered on a three-point Likert scale (1 = “*hardly ever or never*,” 2 = “*some of the time*,” and 3 = “*often*”): “How much of the time do you feel you lack companionship?,” “How much of the time do you feel left out?,” and “How much of the time do you feel isolated from others?” A sum score was built across the items, leading to a loneliness indicator ranging from 3 “*not lonely*” to 9 “*very lonely*.” The three-item loneliness scale has been found to have satisfactory reliability and both concurrent and discriminant validity in older adults (Hughes et al., 2004).

Depressive symptoms were assessed through the EURO-D scale including 12 items: depressed mood, pessimism, wishing for death, guilt, sleep, interest, irritability, appetite, fatigue, concentration, enjoyment, and tearfulness (Copeland et al., 2004; Prince et al., 1999). With each item coded as 0 (symptom absent) and 1 (symptom present), a sum score was generated ranging from 0 to 12, with higher values indicating more depressive symptoms present in the respondent (Boisgontier et al., 2020). The EURO-D scale has been reported to have adequate reliability (Courtin et al., 2015; Portellano-Ortiz et al., 2018; Tomás et al., 2022) and good psychometric properties as an indicator of depression symptomatology in adults aged 50 years and older (Tomás et al., 2022).

### Outcome: Quality of Life

The QoL scale used was the revised version of CASP-19, a theoretically grounded measure of the QoL in older age (Hyde et al., 2003). The revised CASP-12 scale includes 12 items in four subscales: control, autonomy, self-realization, and pleasure (see Supplementary Material for individual items). Each item was assessed on a four-point Likert scale (1 = “*often*,” 2 = “*sometimes*,” 3 = “*rarely*,” and 4 = “*never*”). The resulting score is the sum across the 12 items and ranges from 12 to 48, with higher numbers indicating higher QoL. In the present study, QoL was assessed across Waves 6–8 (Supplementary Figure 2).

### Confounders

Variables that are known to confound the exposure–mediator, mediator–outcome, and exposure–outcome associations were identified based on existing literature (Makovski et al., 2019). As time-invariant confounders gender, and highest educational attainment (primary, secondary, and tertiary) according to the International Standard Classification of Educational Degrees were included. The time-varying confounders were assessed simultaneously with the exposure and included age, country of residence, employment status (employed, unemployed, retired, and out of the labor force), partnership status (alone and partnered), household financial strain, which was based on whether the respondents were able to make ends meet (*with great difficulty, with some difficulty, fairly easily*, and *easily*), pain (“Are you troubled with pain,” no, mild, moderate, and severe), and number of observations per respondent to account for selection effects.

### Statistical Analysis

Model-based causal mediation analyses that draw on the counterfactual framework were conducted by using the *mediation* package in R (Tingley et al., 2014). This package allows us to decompose the overall association between multimorbidity and QoL into causal direct and indirect effects with respect to the mediators under the assumption of sequential ignorability. Aiming at minimizing reverse causation bias, the examined exposure, mediators, and outcome variables were temporally separated (Supplementary Figure 2). Thus, the exposure at Wave *W* (i.e., multimorbidity) was hypothesized to be associated with mediators at *W* + 1 (i.e., ADL, IADL, loneliness, and depressive symptoms) and outcome at *W* + 2 (i.e., QoL), with an approximate interval of 2 years between each wave. Despite this modeling strategy temporally separating the variables, the sequential ignorability assumption may not hold completely in this observational study, which can limit the causal inference drawn from the results (Baranyi et al., 2022; Tingley et al., 2014).

The analyses were completed in three steps, consistent with past literature using a similar approach (Baranyi et al., 2022). First, a mediator model was estimated by regressing the mediator (i.e., ADL, IADL, loneliness, and depressive symptoms) on the exposure (i.e., multimorbidity) and covariates applying linear mixed-effects models nesting observations (Level 1) within individuals (Level 2), with a random intercept at Level 2. Second, an outcome model was estimated by regressing QoL on the exposure, mediator, and covariates applying linear mixed-effects models nesting observations (Level 1) within individuals (Level 2), with a random intercept at Level 2. Third, the mediator and outcome models were used to test the direct and indirect effects including 95% confidence intervals (CIs) by running 1,000 quasi-Bayesian Monte Carlo simulations (Tingley et al., 2014).

The mediator and outcome models were estimated separately for each mediator, including the same set of covariates. Specifically, a model without including the other mediators was first tested (unadjusted mediation). Subsequently, a model adjusting for the other mediators to examine mediation effects above and beyond the other mediators was tested (adjusted mediation). The models allowed reporting the percentage mediated, indicating the share of the total effect accounted for by the respective mediator.

Moderated mediation was performed to examine whether the mediation pathways differed according to age, gender, educational attainment, and household financial strain. Existing research suggested that disease burden and needs in people living with multimorbidity may vary considerably across different socioeconomic groups (Barnett et al., 2012; Makovski et al., 2019). The moderated mediation was tested by including interaction terms between moderator and mediator in the fully adjusted (including all mediators) mediator and outcome models and by computing the mediation function for each level of the moderator (Baranyi et al., 2022; Tingley et al., 2014).

Two sensitivity analyses were performed: (1) Acknowledging that prior studies have demonstrated notable links between loneliness and depression (Cacioppo et al., 2006), follow-up analyses were performed in which models excluding either depressive symptoms or loneliness from the list of covariates were tested. Thus, in the adjusted mediation models, the mediation pathways for loneliness and depressive symptoms were examined while not adjusting for the other mediator, respectively (partly adjusted mediation with ADL and IADL only). (2) To assess whether the findings were mainly driven by individuals with depression at baseline, the mediation pathways were tested on a subsample excluding respondents with depression, indicated by a score of 4 or higher on the EURO-D scale (Prince et al., 1999).

## Results

### Descriptive Results

Supplementary Table 1 shows the descriptive statistics of the 36,908 individuals at baseline stratified by respondents with (two or more chronic conditions) and without multimorbidity (zero or one chronic condition). Simple association tests revealed that participants with multimorbidity were older, female, lived less often in a couple, were more troubled by pain, had lower educational attainment, greater household financial strain, had limitations with ADL and IADL, felt lonelier, had more depressive symptoms, and lower QoL (*p* < .001). Due to missing data and the analytical approach including time lags between exposure, mediators, and outcome, 26.8% of respondents were excluded from the analyses (Supplementary Figure 3 for flowchart of participant inclusion and Supplementary Table 7 for baseline comparison of included and excluded individuals).

### Unadjusted Mediation

The results of the mediation analyses adjusting for covariates but not for the other mediators revealed that multimorbidity was significantly associated with lower QoL (Table 1). The larger proportion of the total association was direct, with differences between −0.86 and −0.73 on the QoL scale for respondents with multimorbidity compared to respondents without multimorbidity depending on the mediator model, while the mediators accounted for varying shares of the total effects (indirect pathway). The indirect pathway from multimorbidity to QoL through ADL limitations accounted for 7.17% of the respective total effect (*b* = −0.06, 95% CI: −0.08 to −0.05, *p* < .001), IADL limitations for 9.96% (*b* = −0.09, 95% CI: −0.10 to −0.08, *p* < .001), loneliness for 7.73% (*b* = −0.07, 95% CI: −0.09 to −0.06, *p* < .001), and depressive symptoms for 23.10% (*b* −0.22, 95% CI: −0.24 to −0.19, *p* < .001).

**Table 1.** Models Linking Multimorbidity and QoL: Unadjusted (for Other Mediators) and Adjusted Longitudinal Mediation Analysis Per Mediator

Adjusted for other mediators?	Mediator	Total effect (*b*)			Direct effect (*b*)			Indirect effect (*b*)			% mediated			
			95% CI			95% CI			95% CI			95% CI		
		Estimate	LL	UL	Estimate	LL	UL	Estimate	LL	UL	Estimate	LL	UL	*p* Value
Unadjusted	ADL	−0.90	−0.98	−0.81	−0.83	−0.92	−0.75	−0.06	−0.08	−0.05	7.17	5.93	9.00	<.001
	IADL	−0.91	−1.00	−0.82	−0.82	−0.91	−0.73	−0.09	−0.10	−0.08	9.96	8.49	12.00	<.001
	Loneliness	−0.93	−1.01	−0.84	−0.86	−0.94	−0.77	−0.07	−0.09	−0.06	7.73	6.02	10.00	<.001
	Depressive symptoms	−0.94	−1.04	−0.86	−0.73	−0.82	−0.64	−0.22	−0.24	−0.19	23.10	20.30	26.00	<.001
Adjusted	ADL	−0.67	−0.75	−0.58	−0.66	−0.74	−0.58	−0.01	−0.01	0.00	0.97	0.56	1.00	<.001
	IADL	−0.68	−0.77	−0.59	−0.66	−0.74	−0.57	−0.02	−0.03	−0.02	3.24	2.30	4.00	<.001
	Loneliness	−0.66	−0.75	−0.57	−0.66	−0.75	−0.57	−0.00	−0.02	0.01	0.41	−1.33	2.00	.650
	Depressive symptoms	−0.79	−0.88	−0.71	−0.66	−0.75	−0.58	−0.13	−0.15	−0.12	16.70	14.40	19.00	<.001
Partly adjusted (with ADL, IADL)	Loneliness	−0.83	−0.91	−0.74	−0.78	−0.86	−0.69	−0.05	−0.07	−0.03	5.89	3.83	8.00	<.001
	Depressive symptoms	−0.84	−0.93	−0.75	−0.66	−0.75	−0.58	−0.17	−0.19	−0.15	20.60	17.80	24.00	<.001

*Notes*: ADL = activities of daily living; *b* = coefficient; 95% CI = 95% confidence interval; IADL = instrumental activities of daily living; LL = lower limit; QoL = quality of life; UL = upper limit.

### Adjusted Mediation

The results of the mediation analyses adjusting for covariates and the other mediators showed that multimorbidity was significantly associated with lower QoL (Table 1). Although the larger proportion of the effect was direct, with a difference of −0.66 on the QoL scale for respondents with multimorbidity compared to respondents without multimorbidity, significant indirect effects through ADL limitations, IADL limitations, and depressive symptoms pathways were found. The ADL limitations accounted for 0.97% (*b* = −0.01, 95% CI: −0.01 to 0.00, *p* < .001) of the respective total effect, IADL limitations for 3.24% (*b* = −0.02, 95% CI: −0.03 to −0.02, *p* < .001), and depressive symptoms for 16.70% (*b* = −0.13, 95% CI: −0.15 to −0.12, *p* < .001). Loneliness did not significantly mediate the association between multimorbidity and QoL (*b* = −0.00, 95% CI: −0.02 to 0.01, *p* = .650).

### Moderated Mediation

**Figure 1.**
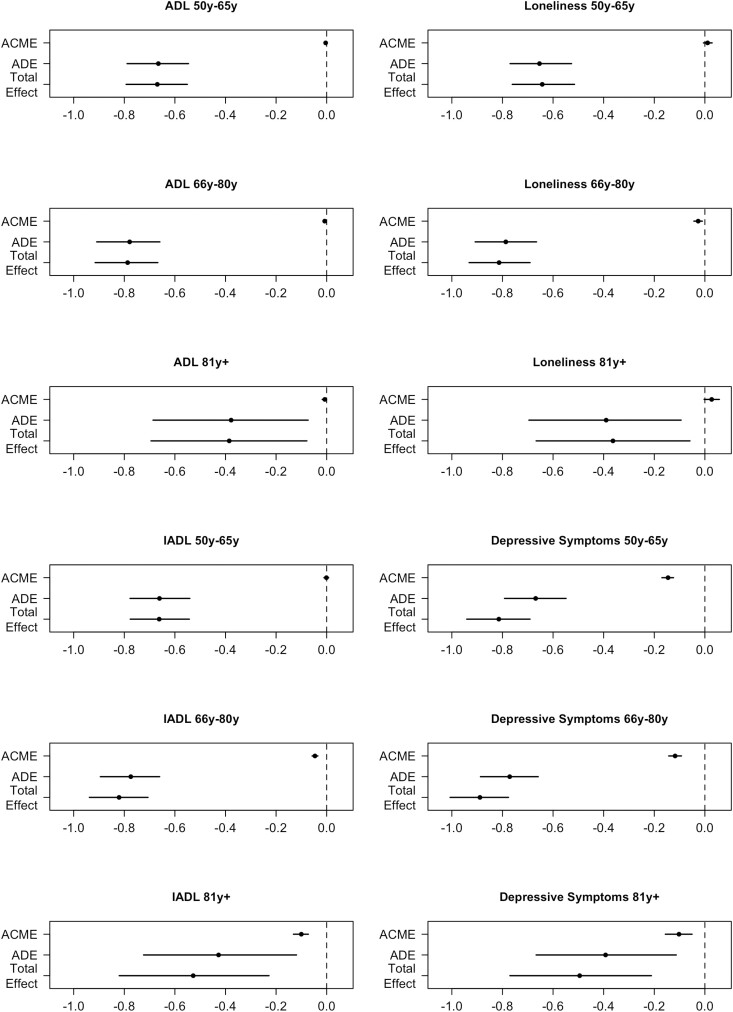
Moderated mediation per mediator of the multimorbidity–QoL association by age categories. ACME = average causal mediation effects; ADE = average direct effects; QoL = quality of life.

By testing a set of models with moderated mediations, it was investigated whether the mediation pathways varied in strength due to different moderators such as age groups (i.e., 50–65 years, 66–80 years, and 81+ years), gender, educational attainment, and household financial strain. For the age groups, the direct effects between multimorbidity and QoL were weaker in the oldest age group with bigger CIs as compared to the two younger age groups (Figure 1), while the indirect effects were similar across age groups (except for loneliness). Consequently, IADL limitations (50–65 years: 0.15%, 66–80 years: 5.56%, 81+ years: 18.90%) and depressive symptoms (50–65 years: 17.90%, 66–80 years: 13.30%, 81+ years: 20.44%) accounted for higher percentages of the association between multimorbidity and QoL in the oldest age group as compared to the two younger age groups (Supplementary Table 2). For loneliness, a significant mediation in the middle (66–80 years) age group was found (3.27%, *b* = −0.03, 95% CI: −0.04 to −0.01, *p* < .001), while the indirect pathway was not significant in the other age groups. For ADL limitations, the indirect pathway was significant in the youngest (50–65 years) age group (0.59%, *b* = −0.00, 95% CI: −0.01 to 0.00, *p* < .002) and middle (66–80 years) age group (0.99%, *b* = −0.01, 95% CI: −0.01 to −0.00, *p* < .001), but not in the oldest age group.

**Figure 2.**
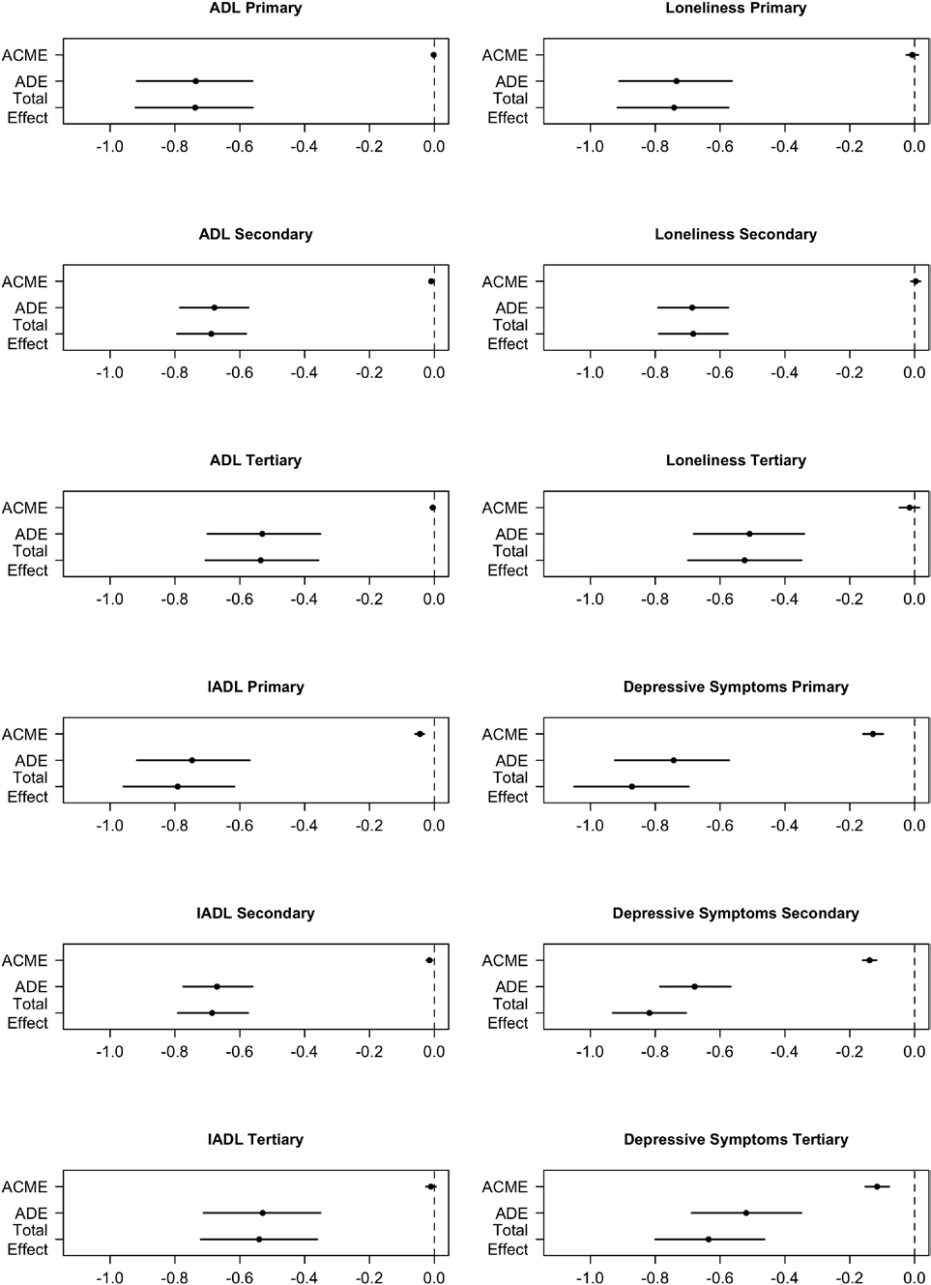
Moderated mediation per mediator of the multimorbidity–QoL association by educational attainment categories. ACME = average causal mediation effects; ADE = average direct effects; QoL = quality of life.

Concerning educational attainment, results indicated that the direct effects between multimorbidity and QoL were weaker in individuals with tertiary education as compared to individuals with primary and secondary education (Figure 2), whereas the indirect effects through the mediators are similar across educational levels. Consequently, ADL limitations (primary: 0.25%, secondary: 1.39%, and tertiary: 2.21%) and depressive symptoms (primary: 14.80%, secondary: 17.00%, and tertiary: 18.20%) accounted for higher percentages of the association between multimorbidity and QoL with increasing educational attainment levels (Supplementary Table 3). The IADL limitations accounted for lower percentages of the association with increasing educational attainment (primary: 5.61%, secondary: 2.21%, and tertiary: 2.01%). The indirect pathway through loneliness was not significant across educational levels.

**Figure 3.**
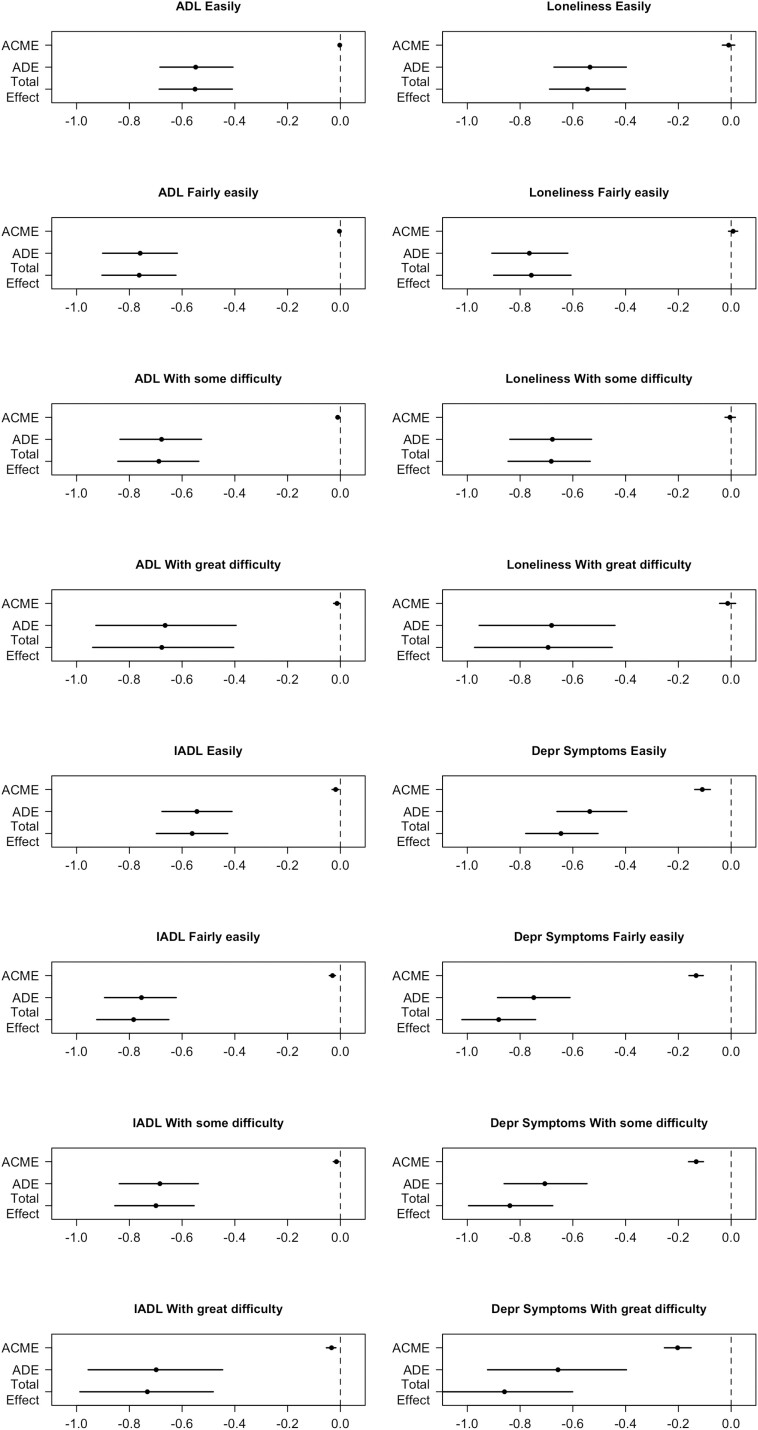
Moderated mediation per mediator of the multimorbidity–-QoL association by household financial strain categories. *Note*: ACME = average causal mediation effects; ADE = average direct effects; QoL = quality of life.

For the different levels of household financial strain, results indicated that ADL limitations significantly accounted for higher percentages across individuals with more financial strain (“*easily*” 0.42%, “*fairly easily*” 0.44%, “*with some difficulty*” 1.44%, and “*with great difficulty*” 1.87%) of the association between multimorbidity and QoL (Supplementary Table 4). Mediation patterns through IADL limitations were not different across levels of household financial strain (Figure 3). Depressive symptoms accounted for significantly higher percentages of the multimorbidity and QoL association in individuals who made ends meet with great difficulty (23.70%) as compared to lower levels of financial strain (*easily*: 17.00%, *fairly easily*: 15.10%, *with some difficulty*: 15.80%; Supplementary Table 4; Figure 3). The indirect pathway through loneliness was not significant across levels of financial strain.

**Figure 4.**
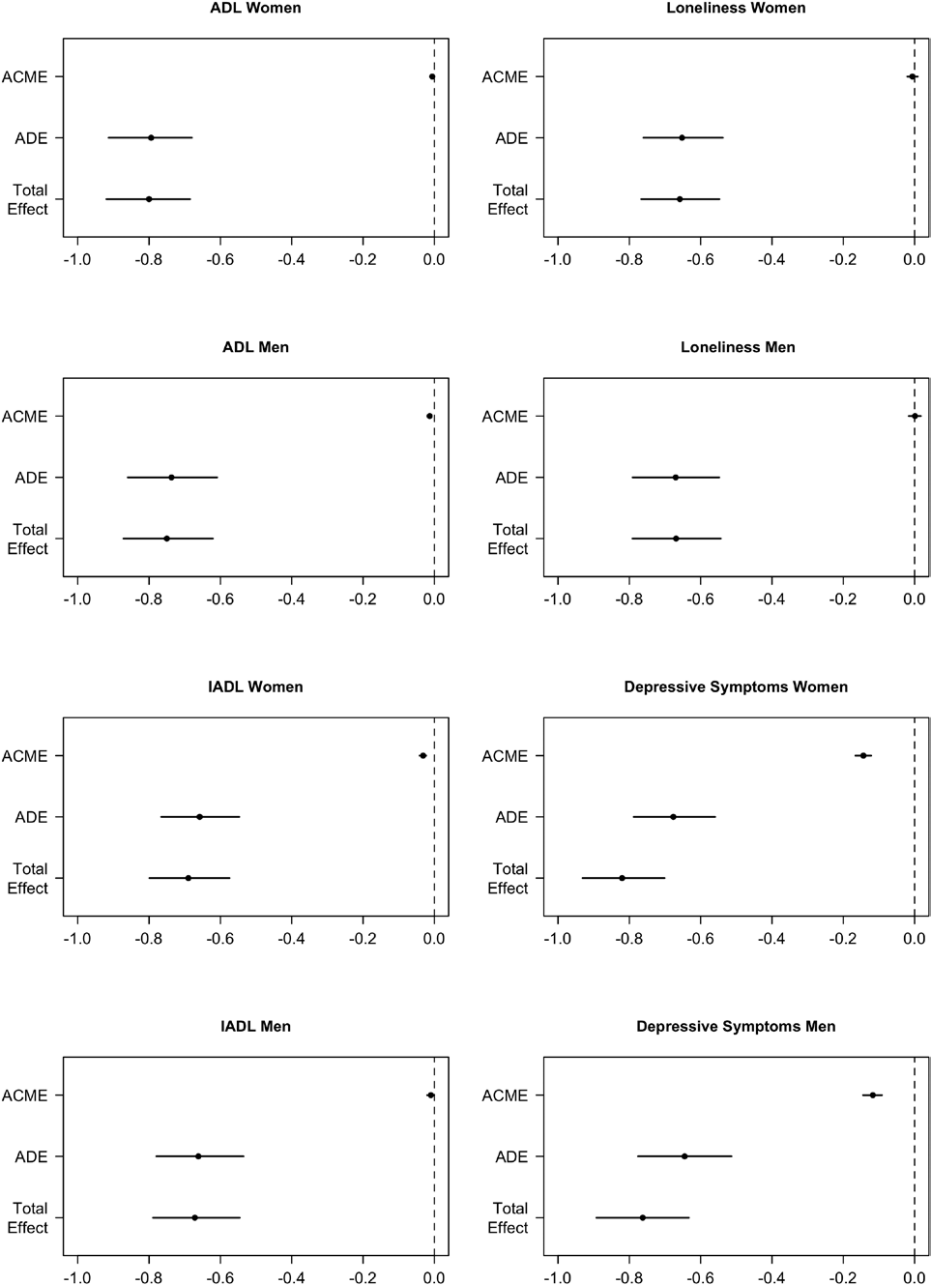
Moderated mediation per mediator of the multimorbidity–QoL association by gender. *Note*: ACME = average causal mediation effects; ADE = average direct effects; QoL = quality of life.

Although multimorbidity prevalence differed between men and women (Supplementary Table 1) and previous research has shown that multimorbidity patterns vary across gender (Abad-Díez et al., 2014), Supplementary Table 5 and Figure 4 reveal that gender did not significantly moderate the mediation of the association between multimorbidity and QoL through limitations with ADL, loneliness, and depressive symptoms. IADL limitations accounted for a higher percentage of the association in women (4.53%) than in men (1.40%).

### Sensitivity Analyses

In the first sensitivity analysis, the loneliness and depressive symptoms pathways were further examined by only adjusting them for ADL and IADL (Table 1, “*Partly adjusted*”). Thus, the loneliness model was partly adjusted for covariates and the mediators limitations with ADL and IADL, but not depressive symptoms, to examine whether depressive symptoms were responsible for the nonsignificant mediation between multimorbidity and QoL through loneliness (Table 1). The results revealed that in the model without depressive symptoms, loneliness significantly accounted for 6.95% of this association (*b* = −0.067, 95% CI: −0.084 to −0.050, *p* < .001). In contrast, depressive symptoms accounted for a higher percentage (24.4%) of the association between multimorbidity and QoL when the model was adjusted for covariates and the mediators ADL and IADL limitations, but not loneliness (*b* = −0.238, 95% CI: −0.259 to −0.220, *p* < .001).

In the second sensitivity analysis, a subsample excluding depressed respondents at baseline was examined (Supplementary Table 6). The results revealed that although the mediation pathway through depressive symptoms was still the most important, it became less strong by approximately 5 percentage points. The other pathways remained virtually unchanged.

## Discussion and Implications

This study set out to examine the modifiable mediators in the pathway between multimorbidity and QoL. The aim was to put intermediary factors in the spotlight that may help to maintain or increase the QoL of individuals living with multimorbidity to improve their successful aging perspectives. This is one of the few studies documenting that multimorbidity is longitudinally associated with lower QoL four years later, using a large-scale study conducted among European adults aged 50 years and older. Individuals with multimorbidity had lower QoL as compared to individuals without multimorbidity by 0.66 points on the scale. This is in line with several systematic reviews, which, however, predominantly analyze cross-sectional studies (Kanesarajah et al., 2018; Makovski et al., 2019; Marengoni et al., 2011). Thus, this study adds to the scarce literature using longitudinal approaches to assess the link between multimorbidity and QoL, which has been identified as crucial in clarifying this link (Makovski et al., 2019).

As hypothesized, the association between multimorbidity and QoL was mediated by limitations in ADL, IADL, and depressive symptoms. Loneliness, however, was not found to mediate this association above and beyond depressive symptoms, indicating that loneliness and depressive symptoms are related to each other, which is in line with previous cross-sectional studies (Stickley & Koyanagi, 2018; Wicke et al., 2014). The mediation effects of ADL and IADL limitations, and depressive symptoms are in line with previous research (Barile et al., 2012; Makovski et al., 2020; She et al., 2019). A cross-sectional study using Wave 6 of the SHARE data, found that the number of symptoms, ADL/IADL limitations, loneliness, and polypharmacy accounted for 69% of the association between the number of diseases and QoL (Makovski et al., 2020). Notably, this study did not perform a thorough mediation analysis but came to this conclusion by comparing the coefficient of the number of diseases on QoL before and after including the predictors. Furthermore, Makovski et al. (2020) showed that loneliness weakened the association between the number of diseases and QoL by 21.5%, while no significant mediation by loneliness was found in the present study. However, Makovski et al. (2020) did not include depressive symptoms in their analyses, which have been shown to offset the loneliness effects in our study.

One sixth of the effect of multimorbidity on QoL was explained by depressive symptoms, whereas functional limitations (ADL/IADL) accounted for less than one twentieth of the effect. A prior cross-sectional study concluded that ADL limitations and depressive symptoms both explained about one fifth of the effect of multimorbidity on health-related QoL (She et al., 2019). Although the findings for depressive symptoms are similar, smaller mediation effects for functional limitations were found, which may be due to study design (longitudinal vs cross-sectional), or, more likely, the chosen QoL outcome (general QoL vs health-related QoL).

Depressive symptoms explained the largest part of the negative effect of multimorbidity on QoL among the mediators investigated, which may contribute to the design of future care programs for people with multimorbidity. Although ADL and IADL limitations should not be disregarded, depressive symptoms may play an important role in the QoL of individuals with multimorbidity, making them a priority when aiming at the maintenance or improvement of QoL in patients living with multimorbidity.

The mediation patterns varied across age categories, level of educational attainment, financial household strain, and gender. Mediations through IADL limitations and depressive symptoms were strongest in the oldest 81+ years age category, indicating that these pathways become especially important in very old age, jointly accounting for about 40% of the negative impact of multimorbidity on QoL. These findings suggest that care programs for individuals living with multimorbidity should put IADL limitations and depressive symptoms at the center of attention when considering people older than 80 years. The increasing importance of the depressive symptoms pathway may be due to the age-related increase in dementia prevalence. Dementia has been shown to manifest as depression in prediagnostic years and is associated with lower QoL (Singh-Manoux et al., 2017). Contrary to the hypothesis, ADL limitations explained the association between multimorbidity and QoL only up to the age of 80 years. The difference in mediation patterns between ADL and IADL may be due to their diverse characteristics. The ADL consist of basic, mostly physical activities that are essential to independent living, whereas performing IADL often additionally requires relatively sophisticated cognition. Limitations with the physical ADL may be more affecting on QoL at a younger age, as they come more unexpectedly (Landös et al., 2019). In addition, expectations of patients with multimorbidity toward QoL can decrease as age increases (Makovski et al., 2019; N’Goran et al., 2017).

Loneliness has been found to significantly mediate the association between multimorbidity and QoL above and beyond depressive symptoms among individuals aged 66–80 years. A potential explanation for this finding is that, on the one hand, in this age bracket (third age) many sudden changes linked to loneliness happen, such as retirement (loss of social contacts at work) and losing friends and family members to death (Segel-Karpas et al., 2018; Yang & Victor, 2011). On the other hand, people often enter a phase of being active with many social activities after retirement (Henning et al., 2016). Individuals with multimorbidity may not be able to experience this post retirement phase the same way as their healthy peers, which negatively affects their QoL. At older ages, loneliness might not have the same impact on QoL as people tend to feel that their social circumstances compare favorably in terms of earlier expectations or relative to peers (Dykstra, 2009).

In line with the hypothesis, ADL and depressive symptoms accounted for higher shares of the association between multimorbidity and QoL with increasing education. This may be due to multimorbidity being more common among individuals with lower education (Makovski et al., 2020; Pathirana & Jackson, 2018). Thus, people with higher education may not expect to live with multimorbidity as their peers are relatively healthier than people with lower education and, consequently, experience a bigger impact on QoL. Contrary to the hypothesis, the IADL mediation pathway became less important with higher educational levels. This may be due to higher cognitive reserves among individuals with higher education, which may support them in the IADL that require cognitive capacities (Duda et al., 2014).

In line with the hypothesis, depressive symptoms accounted for almost one fourth of the association between multimorbidity and QoL among individuals with the greatest difficulty making ends meet (household financial strain). This suggests that depressive symptoms in people with multimorbidity play an especially important role if they live in households with greater difficulties making ends meet. It could be that financially vulnerable individuals with multimorbidity worry a great deal about adequate provision of care and medications beacuse multimorbidity may make it impossible to work and treatment may be costly (Larkin et al., 2021). Thus, in order to best support individuals with multimorbidity, one important policy implication of this study is that health systems should enable affordable care for multimorbidity patients, especially those coming from financially disadvantaged households, to allow for optimal QoL outcomes.

The hypothesis on moderation effects by gender was only partly confirmed, with stronger mediation effects through IADL for women. The mediation pathways through ADL and depressive symptoms did not differ by gender. Previous studies have come to mixed conclusions regarding gender. Supporting the finding in this study, existing systematic reviews have found that the impact of multimorbidity on QoL does not differ by gender (Fortin et al., 2004; Kanesarajah et al., 2018). However, a recent systematic review found stronger effects for women, but only for measures focused on physical and mental QoL (Makovski et al., 2019). Because research on the multimorbidity experience of men and women is scarce (Makovski et al., 2019), this study provides findings filling this gap, indicating that gender may only influence the IADL pathway between multimorbidity and QoL. This finding suggests that care programs focusing on IADL in people living with multimorbidity should consider gender differences, with more pronounced needs among women.

The strengths of this study include its large European-wide and longitudinal sample allowing for a thorough mediation analysis of the factors that account for the multimorbidity–QoL association. Most of the evidence from prior studies is based on cross-sectional data. Moreover, to the best of our knowledge, no existing study has used moderated mediation methods to examine whether mediation patterns differed due to various sociodemographic moderators, such as age, gender, education, and financial household strain.

Despite these strengths, several limitations are noteworthy. First, information on chronic conditions in the SHARE study is self-reported and based on a list of 20 conditions. Individuals who have chronic conditions not mentioned in this list may have been misclassified. The number of participants with multimorbidity may have been further underestimated in this study because self-reported conditions tend to be fewer as compared to numbers in administrative data (Gruneir et al., 2020). However, we would expect the associations found in this study to be even stronger with higher prevalences of multimorbidity. Second, although longitudinal data was used to chronologically separate multimorbidity, mediators, and QoL, the possibility of reverse causation cannot be fully excluded. For instance, it has been shown that depression and anxiety can lead to higher risks of accumulating chronic conditions (Bobo et al., 2022). Nevertheless, our statistical approach was based on the plausible hypothesis that ADL, IADL, loneliness, and depressive symptoms may be on the causal pathway between multimorbidity and QoL, as suggested by theory and by multiple cross-sectional studies (Barile et al., 2012; Makovski et al., 2020; She et al., 2019). Third, causal mediation analysis requires a set of assumptions (i.e., sequential ignorability) to make valid inferences on direct and indirect effects: no unmeasured confounding in (a) exposure–outcome, (b) mediator–outcome, (c) exposure–mediator relationship, and (d) no mediator–outcome confounder affected by the exposure (Tingley et al., 2014; VanderWeele, 2015). These assumptions may not hold in observational studies and relevant sensitivity analyses to test these assumptions were not available for multilevel models (Baranyi et al., 2022; Tingley et al., 2014), which may limit the causal conclusions in this study. Finally, due to the analytical design of our study, only participants with at least three consecutive observations and full information across follow-up were included. This resulted in a selective sample (Supplementary Figure 3), which may have affected the findings due to survivor and health selection biases (Bhamra et al., 2008; Enzenbach et al., 2019). Specifically, excluded participants due to missing information were less likely to be employed and to experience no pain, and more likely to be living with multimorbidity, feel lonelier, have more ADL and IADL limitations, more depressive symptoms, lower QoL, lower education, and more financial strain than individuals included in the final analyses (*p* < .001; Supplementary Table 7).

In summary, older European individuals living with multiple illnesses experience poorer QoL over time due to their multimorbidity. The extent to which multimorbidity negatively influences QoL depends crucially on their functional limitations (ADL and IADL) as well as depressive symptoms, with the latter being the most important. Furthermore, the importance of these intermediary factors changes according to age, education, and household financial strain, as well as gender to a lesser degree. Because care for patients with multimorbidity is complex and costly, focusing on QoL can be worthwhile as it could help to better address individuals’ needs, optimize the use of care efforts, and minimize associated costs. These findings may increase awareness of the functional and mental health implications of older adults living with multiple diseases in Europe. Additionally, this study points toward the need for designing multidisciplinary care interventions that not only aim at treating multimorbidity but also mental and functional health, while considering differences in age, education, financial resources, and gender. Thus, translating the study findings into concrete actions, such as redirecting care efforts related to multimorbidity to the identified intermediary factors, will lead to a further positive impact on the life of individuals, offering them a successful aging perspective despite health limitations.

## Supplementary Material

igad047_suppl_Supplementary_Material

## Data Availability

This paper uses data from SHARE Waves 4, 5, 6, 7, and 8 (dois: 10.6103/SHARE.w4.800, 10.6103/SHARE.w5.800, 10.6103/SHARE.w6.800, 10.6103/SHARE.w7.800, 10.6103/SHARE.w8.800), see Börsch-Supan et al. (2013) for methodological details. The SHARE data collection has been funded by the European Commission, Directorate-General for Research and Innovation through FP5 (QLK6-CT-2001-00360), FP6 (SHARE-I3: RII-CT-2006-062193, COMPARE: CIT5-CT-2005-028857, SHARELIFE: CIT4-CT-2006-028812), FP7 (SHARE-PREP: GA N°211909, SHARE-LEAP: GA N°227822, SHARE M4: GA N°261982, DASISH: GA N°283646) and Horizon 2020 (SHARE-DEV3: GA N°676536, SHARE-COHESION: GA N°870628, SERISS: GA N°654221, SSHOC: GA N°823782, SHARE-COVID19: GA N°101015924) and by DG Employment, Social Affairs & Inclusion through VS 2015/0195, VS 2016/0135, VS 2018/0285, VS 2019/0332, and VS 2020/0313. Additional funding from the German Ministry of Education and Research, the Max Planck Society for the Advancement of Science, the U.S. National Institute on Aging (U01_AG09740-13S2, P01_AG005842, P01_AG08291, P30_AG12815, R21_AG025169, Y1-AG-4553-01, IAG_BSR06-11, OGHA_04-064, HHSN271201300071C, RAG052527A) and from various national funding sources is gratefully acknowledged (data can be accessed at www.share-project.org).
