# Supplementary material for: Multimorbidity and Quality of Life: The Mediating Role of ADL, IADL, Loneliness, and Depressive Symptoms
Source: Innov Aging. 2023 Jun 4;7(4):igad047. doi: 10.1093/geroni/igad047 (PMC10332504; doi:10.1093/geroni/igad047)

**Online Supplementary Material**

**Content**

- **Exposure: Multimorbidity**
- **Outcome: Quality of Life (QoL)**
- **Table S1.** Baseline sample characteristics and univariate associations.
- **Table S2.** Models linking multimorbidity and QoL split by age groups: Moderated longitudinal mediation analysis per mediator
- **Table S3.** Models linking multimorbidity and QoL split by educational attainment: Moderated longitudinal mediation analysis per mediator
- **Table S4.** Models linking multimorbidity and QoL split by household financial strain: Moderated longitudinal mediation analysis per mediator
- **Table S5.** Models linking multimorbidity and QoL split by gender: Moderated longitudinal mediation analysis per mediator.
- **Table S7.** Baseline sample characteristics across included and excluded participants.
- **Figure S1.** Illustration of the theoretical model, depicting A) overall association B) mediation pathways and C) moderated mediation. Note: Moderators = age, gender, educational attainment, household financial strain. *a_i_b_i_* = indirect effect through respective mediator, *c’* = direct effect, *c* = *c’* + *a_i_b_i_*, = total effect, *w* = wave of measurement
- **Figure S2.** Longitudinal mediation model over the five survey waves. Mediator = ADL, IADL, loneliness, depressive symptoms. W4-8 = waves of data collection (approx. 2 years apart). QoL = Quality of Life.
- **Figure S3.** Flow chart of participant inclusion. Note that one observation (Obs) here means an observation across three consecutive waves.

**Exposure: Multimorbidity**

Chronic conditions were assessed through the question: “Has a doctor ever told you that you had or do you currently have any of the conditions [listed] on this card?”. The 20 health conditions listed were: heart attack, including myocardial infarction or coronary thrombosis or any other heart problem including congestive heart failure; high blood pressure or hypertension; high blood cholesterol; stroke or cerebral vascular disease; diabetes or high blood sugar; chronic lung disease such as chronic bronchitis or emphysema; asthma; osteoporosis; cancer or malignant tumor, including leukemia or lymphoma but excluding minor skin cancers; stomach or duodenal ulcer, peptic ulcer; Parkinson disease; cataracts; hip fracture; other fractures; Alzheimer's disease, dementia, organic brain syndrome, senility or any other serious memory impairment; benign tumor (fibroma, polypus, angioma); other affective or emotional disorders, including anxiety, nervous or psychiatric problems; rheumatoid arthritis; osteoarthritis, or other rheumatism; chronic kidney disease. For determining multimorbidity, we did not consider cataracts, hip fractures, and other fractures due to their not necessarily chronic nature. Moreover, we did not consider affective or emotional disorders, including anxiety, nervous or psychiatric problems as we were most interested in physical multimorbidity and because we used mental health issues, more specifically, depressive symptoms as a mediator. All conditions were measured in all waves, except for asthma, osteoporosis, and benign tumor, which were collected in wave 4 only. Chronic kidney disease was measured from wave 6 on. Multimorbidity was defined as the coexistence of 2 or more chronic conditions (Demirer et al., 2021; Makovski et al., 2019; Navickas et al., 2016; Rijken et al., 2016), and was assessed in waves 4, 5, and 6 (Figure S1, Supplementary Material).

**References**

Demirer, I., Bethge, M., Spyra, K., Karbach, U., & Pfaff, H. (2021). Does social support mediate the effect of multimorbidity on mental wellbeing in the German working population? A longitudinal mediation analysis using structural equation modelling. SSM - Population Health, 13, 100744. https://doi.org/10.1016/j.ssmph.2021.100744

Makovski, T. T., Schmitz, S., Zeegers, M. P., Stranges, S., & van den Akker, M. (2019). Multimorbidity and quality of life: Systematic literature review and meta-analysis. Ageing Research Reviews, 53, 100903. https://doi.org/10.1016/j.arr.2019.04.005

Navickas, R., Petric, V.-K., Feigl, A. B., & Seychell, M. (2016). Multimorbidity: What Do We Know? What Should We Do? Journal of Comorbidity, 6(1), 4–11. https://doi.org/10.15256/joc.2016.6.72

Rijken, M., Struckmann, V., van der Heide, I., Hujala, A., Barbabella, F., van Ginneken, E., & Schellevis, F. (2016). How to improve care for people with multimorbidity in Europe? (Health Systems and Policy Analysis) [Policy Brief]. WHO Regional Office for Europe. https://www.euro.who.int/__data/assets/pdf_file/0004/337585/PB_23.pdf

**Quality of Life (QoL)**

The CASP-12 is a theoretically grounded measure of QoL in older ages composed of 12 items in four subscales.

| **Subscale** | **Item** |
| --- | --- |
| Control | How often do you think your age prevents you from doing the things you would like to do? |
| Control | How often do you feel that what happens to you is out of your control? |
| Control | How often do you feel left out of things? |
| Autonomy | How often do you think that you can do the things that you want to do? |
| Autonomy | How often do you think that family responsibilities prevent you from doing what you want to do? |
| Autonomy | How often do you think that shortage of money stops you from doing the things you want to do? |
| Pleasure | How often do you look forward to each day? |
| Pleasure | How often do you feel that your life has meaning? |
| Pleasure | How often, on balance, do you look back on your life with a sense of happiness? |
| Self-Realization | How often do you feel full of energy these days? |
| Self-Realization | How often do you feel that life is full of opportunities? |
| Self-Realization | How often do you feel that the future looks good for you? |

**Table S1.** Baseline sample characteristics and univariate associations.

| **Variable** | **No multimorbidity**  **N = 23,022** | **Multimorbidity**  **N = 13,886** | **P-Value** |
| --- | --- | --- | --- |
|  | **Mean (SD)** | **Mean (SD)** |  |
| Age | 63.6 (8.9) | 68.0 (8.9) | <.001 |
| Gender (N, %) |  |  | <.001 |
| Man | 10,264 (44.6%) | 5,762 (41.5%) |  |
| Woman | 12,758 (55.4%) | 8,124 (58.5%) |  |
| Observations^a^ | 1.7 (0.7) | 1.7 (0.7) | 0.033 |
| ADL | 0.1 (0.5) | 0.3 (0.9) | <.001 |
| IADL | 0.2 (0.7) | 0.5 (1.2) | <.001 |
| Loneliness | 3.7 (1.2) | 4.0 (1.4) | <.001 |
| Depressive symptoms | 1.9 (1.9) | 2.8 (2.3) | <.001 |
| QoL | 38.8 (5.7) | 36.2 (6.4) | <.001 |
| Education (N, %) |  |  | <.001 |
| Primary | 3,688 (16%) | 3,329 (24%) |  |
| Secondary | 13,040 (56.6%) | 7,818 (56.3%) |  |
| Tertiary | 6,294 (27.3%) | 2,739 (19.7%) |  |
| Household financial strain (N, %) |  |  | <.001 |
| Easily | 8,976 (39%) | 4,237 (30.5%) |  |
| Fairly easily | 7,321 (31.8%) | 4,214 (30.3%) |  |
| With some difficulty | 5,162 (22.4%) | 3,898 (28.1%) |  |
| With great difficulty | 1,563 (6.8%) | 1,537 (11.1%) |  |
| Employment status (N, %) |  |  | <.001 |
| Employed | 8,755 (38%) | 2,333 (16.8%) |  |
| Out of the labour force | 2,101 (9.1%) | 1,852 (13.3%) |  |
| Retired | 11,370 (49.4%) | 9,340 (67.3%) |  |
| Unemployed | 796 (3.5%) | 361 (2.6%) |  |
| Partnership status (N, %) |  |  | <.001 |
| In couple | 16,778 (72.9%) | 9,510 (68.5%) |  |
| Alone | 6,244 (27.1%) | 4,376 (31.5%) |  |
| Pain level (N, %) |  |  | <.001 |
| No | 14,867 (64.6%) | 5,771 (41.6%) |  |
| Mild | 2,390 (10.4%) | 1,447 (10.4%) |  |
| Moderate | 4,406 (19.1%) | 4,502 (32.4%) |  |
| Severe | 1,359 (5.9%) | 2,166 (15.6%) |  |
| Country (N, %) |  |  | <.001 |
| Austria | 1,693 (7.4%) | 944 (6.8%) |  |
| Belgium | 2,181 (9.5%) | 1,419 (10.2%) |  |
| Czech Republic | 1,906 (8.3%) | 1,487 (10.7%) |  |
| Denmark | 1,857 (8.1%) | 905 (6.5%) |  |
| Estonia | 2,107 (9.2%) | 1,740 (12.5%) |  |
| France | 1,698 (7.4%) | 1,007 (7.3%) |  |
| Germany | 1,953 (8.5%) | 1,298 (9.3%) |  |
| Israel | 441 (1.9%) | 316 (2.3%) |  |
| Italy | 1,905 (8.3%) | 1,089 (7.8%) |  |
| Luxembourg | 360 (1.6%) | 310 (2.2%) |  |
| Slovenia | 1,300 (5.6%) | 674 (4.9%) |  |
| Spain | 2,040 (8.9%) | 1,312 (9.4%) |  |
| Sweden | 1,787 (7.8%) | 825 (5.9%) |  |
| Switzerland | 1,794 (7.8%) | 560 (4%) |  |

Note. ^a^ One observation corresponds to an observation across 3 consecutive waves, ADL = Activities of daily living. IADL = Instrumental activities of daily living. QoL = quality of life. *SD*: standard deviation. P-values of univariate associations with multimorbidity were computed using two-sided T-tests and chi-square tests (*χ2*) for continuous and categorical variables, respectively.

**Table S2.** Models linking multimorbidity and QoL split by age groups: Moderated longitudinal mediation analysis per mediator

| Mediator | Moderator level | Total effect (*b*) | | |  | Direct effect (*b*) | | |  | Indirect effect (*b*) | | |  | % mediated | | | |
| --- | --- | --- | --- | --- | --- | --- | --- | --- | --- | --- | --- | --- | --- | --- | --- | --- | --- |
|  |  |  | 95% CI | |  |  | 95% CI | |  |  | 95% CI | |  |  | 95% CI | |  |
|  |  | Estimate | LL | UL |  | Estimate | LL | UL |  | Estimate | LL | UL |  | Estimate | LL | UL | P- value |
| ADL | -65y | -0.67 | -0.79 | -0.55 |  | -0.67 | -0.79 | -0.55 |  | -0.00 | -0.01 | 0.00 |  | 0.59 | 0.16 | 1.00 | 0.002 |
|  | 66y-80y | -0.79 | -0.92 | -0.67 |  | -0.78 | -0.91 | -0.66 |  | -0.01 | -0.01 | -0.00 |  | 0.99 | 0.46 | 2.00 | <0.001 |
|  | 81y+ | -0.39 | -0.69 | -0.08 |  | -0.38 | -0.69 | -0.07 |  | -0.01 | -0.02 | 0.00 |  | 1.84 | -0.06 | 19.00 | 0.054 |
| IADL | -65y | -0.66 | -0.78 | -0.54 |  | -0.66 | -0.78 | -0.54 |  | -0.00 | -0.01 | 0.01 |  | 0.15 | -1.17 | 2.00 | 0.830 |
|  | 66y-80y | -0.82 | -0.94 | -0.71 |  | -0.77 | -0.90 | -0.66 |  | -0.05 | -0.06 | -0.03 |  | 5.65 | 4.10 | 7.00 | <0.001 |
|  | 81y+ | -0.53 | -0.82 | -0.23 |  | -0.43 | -0.72 | -0.12 |  | -0.10 | -0.13 | -0.07 |  | 18.90 | 10.60 | 46.00 | <0.001 |
| Loneliness | -65y | -0.64 | -0.76 | -0.52 |  | -0.65 | -0.77 | -0.53 |  | 0.01 | -0.01 | 0.03 |  | -1.70 | -4.71 | 1.00 | 0.230 |
|  | 66y-80y | -0.81 | -0.93 | -0.69 |  | -0.79 | -0.91 | -0.67 |  | -0.03 | -0.04 | -0.01 |  | 3.27 | 1.23 | 6.00 | <0.001 |
|  | 81y+ | -0.36 | -0.67 | -0.06 |  | -0.39 | -0.70 | -0.09 |  | 0.03 | -0.00 | 0.06 |  | -6.98 | -42.05 | 1.00 | 0.100 |
| Depressive symptoms | -65y | -0.81 | -0.94 | -0.69 |  | -0.67 | -0.79 | -0.55 |  | -0.15 | -0.17 | -0.12 |  | 17.90 | 14.50 | 22.00 | <0.001 |
|  | 66y-80y | -0.89 | -1.01 | -0.78 |  | -0.77 | -0.89 | -0.66 |  | -0.12 | -0.14 | -0.09 |  | 13.30 | 10.40 | 16.00 | <0.001 |
|  | 81y+ | -0.50 | -0.77 | -0.22 |  | -0.39 | -0.67 | -0.11 |  | -0.10 | -0.16 | -0.05 |  | 20.44 | 9.96 | 51.00 | <0.001 |

Note. ADL = Activities of daily living, IADL = Instrumental activities of daily living, *b* = coefficient, 95% CI = 95% Confidence Interval, LL = lower limit, UL = upper limit.

**Table S3.** Models linking multimorbidity and QoL split by educational attainment: Moderated longitudinal mediation analysis per mediator

| Mediator | Moderator level | Total effect (*b*) | | |  | Direct effect (*b*) | | |  | Indirect effect (*b*) | | |  | % mediated | | | |
| --- | --- | --- | --- | --- | --- | --- | --- | --- | --- | --- | --- | --- | --- | --- | --- | --- | --- |
|  |  |  | 95% CI | |  |  | 95% CI | |  |  | 95% CI | |  |  | 95% CI | |  |
|  |  | Estimate | LL | UL |  | Estimate | LL | UL |  | Estimate | LL | UL |  | Estimate | LL | UL | P value |
| ADL | Primary | -0.74 | -0.92 | -0.56 |  | -0.74 | -0.92 | -0.56 |  | -0.00 | -0.01 | 0.00 |  | 0.25 | -0.20 | 1.00 | 0.250 |
|  | Secondary | -0.69 | -0.79 | -0.58 |  | -0.68 | -0.79 | -0.57 |  | -0.01 | -0.02 | -0.01 |  | 1.39 | 0.78 | 2.00 | <0.001 |
|  | Tertiary | -0.69 | -0.79 | -0.57 |  | -0.67 | -0.78 | -0.56 |  | -0.02 | -0.03 | 0.01 |  | 2.21 | 0.80 | 4.00 | 0.002 |
| IADL | Primary | -0.79 | -0.96 | -0.62 |  | -0.75 | -0.92 | -0.57 |  | -0.05 | -0.06 | -0.03 |  | 5.61 | 3.84 | 8.00 | 0.006 |
|  | Secondary | -0.69 | -0.79 | -0.57 |  | -0.67 | -0.78 | -0.56 |  | -0.02 | -0.03 | -0.01 |  | 2.21 | 0.80 | 4.00 | 0.002 |
|  | Tertiary | -0.54 | -0.72 | -0.36 |  | -0.53 | -0.71 | -0.35 |  | -0.01 | -0.03 | 0.01 |  | 2.01 | -0.99 | 5.00 | 0.160 |
| Loneliness | Primary | -0.85 | -1.04 | -0.67 |  | -0.84 | -1.02 | -0.66 |  | -0.01 | -0.03 | 0.01 |  | 1.27 | -0.97 | 4.00 | 0.270 |
|  | Secondary | -0.76 | -0.87 | -0.64 |  | -0.76 | -0.87 | -0.65 |  | 0.00 | -0.01 | 0.02 |  | -0.50 | -2.61 | 2.00 | 0.620 |
|  | Tertiary | -0.58 | -0.76 | -0.41 |  | -0.57 | -0.75 | -0.39 |  | -0.01 | -0.05 | 0.01 |  | 2.35 | -2.59 | 8.00 | 0.350 |
| Depressive symptoms | Primary | -0.87 | -1.05 | -0.70 |  | -0.74 | -0.92 | -0.57 |  | -0.13 | -0.16 | -0.10 |  | 14.80 | 10.90 | 20.00 | <0.001 |
|  | Secondary | -0.82 | -0.93 | -0.70 |  | -0.68 | -0.79 | -0.57 |  | -0.14 | -0.16 | -0.12 |  | 17.00 | 14.20 | 21.00 | <0.001 |
|  | Tertiary | -0.64 | -0.80 | -0.46 |  | -0.52 | -0.69 | -0.35 |  | -0.12 | -0.15 | -0.08 |  | 18.20 | 12.00 | 27.00 | <0.001 |

Note. ADL = Activities of daily living, IADL = Instrumental activities of daily living, *b* = coefficient, 95% CI = 95% Confidence Interval, LL = lower limit, UL = upper limit.

**Table S4.** Models linking multimorbidity and QoL split by household financial strain: Moderated longitudinal mediation analysis per mediator

| Mediator | Moderator level | Total effect (*b*) | | |  | Direct effect (*b*) | | |  | Indirect effect (*b*) | | |  | % mediated | | | |
| --- | --- | --- | --- | --- | --- | --- | --- | --- | --- | --- | --- | --- | --- | --- | --- | --- | --- |
|  |  |  | 95% CI | |  |  | 95% CI | |  |  | 95% CI | |  |  | 95% CI | |  |
|  |  | Estimate | Lower | Upper |  | Estimate | Lower | Upper |  | Estimate | Lower | Upper |  | Estimate | Lower | Upper | P value |
| ADL | Easily | -0.55 | -0.69 | -0.41 |  | -0.55 | -0.68 | -0.41 |  | -0.00 | -0.01 | 0.00 |  | 0.42 | -0.59 | 2.00 | 0.410 |
|  | Fairly easily | -0.76 | -0.90 | -0.62 |  | -0.76 | -0.90 | -0.62 |  | -0.00 | -0.01 | 0.00 |  | 0.44 | 0.03 | 1.00 | 0.030 |
|  | With some difficulty | -0.69 | -0.84 | -0.54 |  | -0.68 | -0.84 | -0.53 |  | -0.01 | -0.02 | 0.00 |  | 1.44 | 0.66 | 3.00 | <0.001 |
|  | With great difficulty | -0.68 | -0.94 | -0.40 |  | -0.66 | -0.93 | -0.40 |  | -0.01 | -0.03 | 0.00 |  | 1.87 | 0.24 | 4.00 | 0.022 |
| IADL | Easily | -0.56 | -0.70 | -0.43 |  | -0.54 | -0.68 | -0.41 |  | -0.02 | -0.03 | -0.01 |  | 3.16 | 0.99 | 6.00 | 0.004 |
|  | Fairly easily | -0.78 | -0.92 | -0.65 |  | -0.75 | -0.89 | -0.62 |  | -0.03 | -0.04 | -0.02 |  | 3.79 | 2.36 | 6.00 | <0.001 |
|  | With some difficulty | -0.70 | -0.86 | -0.55 |  | -0.68 | -0.84 | -0.54 |  | -0.02 | -0.03 | -0.01 |  | 2.11 | 0.67 | 4.00 | 0.002 |
|  | With great difficulty | -0.73 | -0.99 | -0.48 |  | -0.70 | -0.96 | -0.45 |  | -0.03 | -0.05 | -0.02 |  | 4.53 | 2.15 | 8.00 | <0.001 |
| Loneliness | Easily | -0.54 | -0.69 | -0.40 |  | -0.54 | -0.67 | -0.40 |  | -0.01 | -0.03 | 0.01 |  | 1.61 | -2.65 | 6.00 | 0.450 |
|  | Fairly easily | -0.76 | -0.90 | -0.61 |  | -0.77 | -0.91 | -0.62 |  | 0.01 | -0.01 | 0.02 |  | -0.99 | -3.52 | 1.00 | 0.410 |
|  | With some difficulty | -0.68 | -0.85 | -0.53 |  | -0.68 | -0.84 | -0.53 |  | -0.00 | -0.02 | 0.02 |  | 0.65 | -2.45 | 3.00 | 0.650 |
|  | With great difficulty | -0.69 | -0.97 | -0.45 |  | -0.68 | -0.96 | -0.44 |  | -0.01 | -0.04 | 0.02 |  | 1.90 | -2.69 | 6.00 | 0.370 |
| Depressive symptoms | Easily | -0.65 | -0.78 | -0.50 |  | -0.54 | -0.66 | -0.40 |  | -0.11 | -0.14 | -0.08 |  | 17.00 | 12.00 | 23.00 | <0.001 |
|  | Fairly easily | -0.88 | -1.02 | -0.74 |  | -0.75 | -0.89 | -0.61 |  | -0.13 | -0.16 | -0.11 |  | 15.10 | 11.90 | 19.00 | <0.001 |
|  | With some difficulty | -0.84 | -1.00 | -0.68 |  | -0.71 | -0.86 | -0.55 |  | -0.13 | -0.16 | -0.10 |  | 15.80 | 12.20 | 21.00 | <0.001 |
|  | With great difficulty | -0.86 | -1.13 | -0.60 |  | -0.66 | -0.92 | -0.40 |  | -0.20 | -0.25 | -0.15 |  | 23.70 | 16.40 | 35.00 | <0.001 |

Note. ADL = Activities of daily living, IADL = Instrumental activities of daily living, *b* = coefficient, 95% CI = 95% Confidence Interval, LL = lower limit, UL = upper limit.

**Table S5.** Models linking multimorbidity and QoL split by gender: Moderated longitudinal mediation analysis per mediator.

| Mediator | Moderator level | Total effect (*b*) | | |  | Direct effect (*b*) | | |  | Indirect effect (*b*) | | |  | % mediated | | | |
| --- | --- | --- | --- | --- | --- | --- | --- | --- | --- | --- | --- | --- | --- | --- | --- | --- | --- |
|  |  |  | 95% CI | |  |  | 95% CI | |  |  | 95% CI | |  |  | 95% CI | |  |
|  |  | Estimate | Lower | Upper |  | Estimate | Lower | Upper |  | Estimate | Lower | Upper |  | Estimate | Lower | Upper | P value |
| ADL | Women | -0.80 | -0.92 | -0.68 |  | -0.79 | -0.91 | -0.68 |  | -0.01 | -0.01 | 0.00 |  | 0.73 | 0.33 | 1.00 | <0.001 |
|  | Men | -0.75 | -0.87 | -0.62 |  | -0.74 | -0.87 | -0.62 |  | -0.01 | -0.02 | -0.01 |  | 1.70 | 0.91 | 3.00 | <0.001 |
| IADL | Women | -0.69 | -0.80 | -0.57 |  | -0.66 | -0.77 | -0.55 |  | -0.03 | -0.04 | -0.02 |  | 4.53 | 3.25 | 6.00 | <0.001 |
|  | Men | -0.67 | -0.79 | -0.55 |  | -0.66 | -0.78 | -0.54 |  | -0.01 | -0.02 | 0.00 |  | 1.40 | 0.06 | 3.00 | 0.044 |
| Loneliness | Women | -0.66 | -0.77 | -0.55 |  | -0.65 | -0.76 | -0.54 |  | -0.00 | -0.02 | 0.01 |  | 0.92 | -1.29 | 3.00 | 0.430 |
|  | Men | -0.67 | -0.79 | -0.54 |  | -0.67 | -0.79 | -0.55 |  | 0.00 | -0.02 | 0.02 |  | -0.18 | -2.87 | 2.00 | 0.900 |
| Depressive symptoms | Women | -0.82 | -0.93 | -0.70 |  | -0.68 | -0.79 | -0.56 |  | -0.14 | -0.17 | -0.12 |  | 17.50 | 14.40 | 22.00 | <0.001 |
|  | Men | -0.76 | -0.89 | -0.63 |  | -0.65 | -0.78 | -0.51 |  | -0.12 | -0.15 | -0.09 |  | 15.30 | 11.80 | 20.00 | <0.001 |

Note. ADL = Activities of daily living, IADL = Instrumental activities of daily living, *b* = coefficient, 95% CI = 95% Confidence Interval, LL = lower limit, UL = upper limit.

**Table S6.** Sensitivity Analysis excluding respondents with depression (EuroD score $\geq$ 4), N = 29,985. Models linking multimorbidity and QoL: Unadjusted (for other mediators) and adjusted longitudinal mediation analysis per mediator

| Adjusted for other mediators? | Mediator | Total effect (*b*) | | |  | Direct effect (*b*) | | |  | Indirect effect (*b*) | | |  | % mediated | | | |  |
| --- | --- | --- | --- | --- | --- | --- | --- | --- | --- | --- | --- | --- | --- | --- | --- | --- | --- | --- |
|  |  |  | 95% CI | |  |  | 95% CI | |  |  | 95% CI | |  |  | 95% CI | |  |  |
|  |  | Estimate | LL | UL |  | Estimate | LL | UL |  | Estimate | LL | UL |  | Estimate | LL | UL | P- value | |
| Unadjusted | ADL | -0.69 | -0.79 | -0.59 |  | -0.64 | -0.74 | -0.55 |  | -0.05 | -0.06 | -0.04 |  | 6.88 | 5.26 | 9.00 | <0.001 | |
|  | IADL | -0.69 | -0.78 | -0.59 |  | -0.62 | -0.71 | -0.52 |  | -0.07 | -0.08 | -0.06 |  | 9.97 | 7.91 | 12.00 | <0.001 | |
|  | Loneliness | -0.70 | -0.79 | -0.61 |  | -0.66 | -0.75 | -0.57 |  | -0.04 | -0.06 | -0.02 |  | 5.59 | 3.17 | 8.00 | <0.001 | |
|  | Depression | -0.71 | -0.81 | -0.61 |  | -0.59 | -0.69 | -0.49 |  | -0.12 | -0.14 | -0.10 |  | 16.50 | 13.40 | 21.00 | <0.001 | |
| Adjusted | ADL | -0.54 | -0.64 | -0.44 |  | -0.54 | -0.64 | -0.44 |  | -0.01 | -0.01 | 0.00 |  | 0.82 | 0.35 | 2.00 | <0.001 | |
|  | IADL | -0.56 | -0.66 | -0.46 |  | -0.54 | -0.64 | -0.45 |  | -0.02 | -0.03 | -0.01 |  | 3.55 | 2.24 | 5.00 | <0.001 | |
|  | Loneliness | -0.54 | -0.64 | -0.45 |  | -0.54 | -0.64 | -0.44 |  | -0.00 | -0.02 | 0.01 |  | 0.32 | -0.64 | 3.00 | 0.790 | |
|  | Depression | -0.61 | -0.71 | -0.52 |  | -0.54 | -0.63 | -0.45 |  | -0.07 | -0.09 | -0.06 |  | 11.56 | 8.89 | 14.00 | <0.001 | |
| Partly adjusted (with ADL, IADL) | Loneliness | -0.63 | -0.72 | -0.52 |  | -0.60 | -0.69 | -0.50 |  | -0.02 | -0.04 | -0.01 |  | 3.92 | 1.18 | 7.00 | 0.002 | |
|  | Depression | -0.63 | -0.72 | -0.53 |  | -0.54 | -0.63 | -0.44 |  | -0.09 | -0.11 | -0.07 |  | 14.42 | 11.31 | 18.00 | <0.001 | |

Note. ADL = Activities of daily living, IADL = Instrumental activities of daily living, *b* = coefficient, 95% CI = 95% Confidence Interval, LL = lower limit, UL = upper limit.

**Table S7.** Baseline sample characteristics across included and excluded participants.

| **Variable** | **Included**  **N = 36908** | **Excluded**  **N = 13484** | **P-Value** |
| --- | --- | --- | --- |
|  | **Mean (SD)** | **Mean (SD)** |  |
| Multimorbidity (N, %) |  |  | <.001 |
| No | 23,022 (62.4%) | 5,551 (41.2%) |  |
| Yes | 13,886 (37.6%) | 4,425 (32.8%) |  |
| NA | 0 (0%) | 3,508 (26%) |  |
| Age | 65.3 (9.2) | 68.1 (11.1) | <.001 |
| NA | 0 (0%) | 3,464 (25.7%) |  |
| Gender (N, %) |  |  | 0.011 |
| Man | 16,026 (43.4%) | 5,683 (42.1%) |  |
| Woman | 20,882 (56.6%) | 7,801 (57.9%) |  |
| NA | 0 (0%) | 0 (0%) |  |
| Observations^a^ | 1.7 (0.7) | 0 (0) | <.001 |
| ADL | 0.2 (0.6) | 0.4 (1.2) | <.001 |
| NA | 0 (0%) | 5,758 (42.7%) |  |
| IADL | 0.3 (0.9) | 0.9 (2.1) | <.001 |
| NA | 0 (0%) | 5,758 (42.7%) |  |
| Loneliness | 3.8 (1.3) | 4.1 (1.5) | <.001 |
| NA | 0 (0%) | 8,140 (60.4%) |  |
| Depressive symptoms | 2.3 (2.1) | 2.6 (2.4) | <.001 |
| NA | 0 (0%) | 8,227 (61.0%) |  |
| QoL | 37.8 (6.1) | 36.3 (6.4) | <.001 |
| NA | 0 (0%) | 8591 (63.7%) |  |
| Education (N, %) |  |  | <.001 |
| Primary | 7,017 (19%) | 3,424 (25.4%) |  |
| Secondary | 20,858 (56.5%) | 7,230 (53.6%) |  |
| Tertiary | 9,033 (24.5%) | 2,504 (18.6%) |  |
| NA | 0 (0%) | 326 (2.4%) |  |
| Household financial strain (N, %) |  |  | <.001 |
| Easily | 13,213 (35.8%) | 2,467 (18.3%) |  |
| Fairly easily | 11,535 (31.3%) | 2,666 (19.8%) |  |
| With some difficulty | 9,060 (24.5%) | 3,104 (23%) |  |
| With great difficulty | 3,100 (8.4%) | 1,572 (11.7%) |  |
| NA | 0 (0%) | 3,675 (27.3%) |  |
| Employment status (N, %) |  |  | <.001 |
| Employed | 11,088 (30%) | 2,106 (15.6%) |  |
| Out of the labour force | 3,953 (10.7%) | 1,439 (10.7%) |  |
| Retired | 20,710 (56.1%) | 5,710 (42.3%) |  |
| Unemployed | 1,157 (3.1%) | 290 (2.2%) |  |
| NA | 0 (0%) | 3,939 (29.2%) |  |
| Partnership status (N, %) |  |  | <.001 |
| In couple | 26,288 (71.2%) | 7,977 (59.2%) |  |
| Alone | 10,620 (28.8%) | 3,647 (27%) |  |
| NA | 0 (0%) | 1,860 (13.8%) |  |
| Pain level (N, %) |  |  | <.001 |
| No | 20,638 (55.9%) | 2,973 (22%) |  |
| Mild | 3,837 (10.4%) | 505 (3.7%) |  |
| Moderate | 8,908 (24.1%) | 1,640 (12.2%) |  |
| Severe | 3,525 (9.6%) | 747 (5.5%) |  |
| NA | 0 (0%) | 7,619 (56.5%) |  |
| Country (N, %) |  |  | <.001 |
| Austria | 2,637 (7.1%) | 413 (3.1%) |  |
| Belgium | 3,600 (9.8%) | 620 (4.6%) |  |
| Czech Republic | 3,393 (9.2%) | 795 (5.9%) |  |
| Denmark | 2,762 (7.5%) | 291 (2.2%) |  |
| Estonia | 3,847 (10.4%) | 898 (6.7%) |  |
| France | 2,705 (7.3%) | 529 (3.9%) |  |
| Germany | 3,251 (8.8%) | 455 (3.4%) |  |
| Israel | 757 (2.1%) | 636 (4.7%) |  |
| Italy | 2,994 (8.1%) | 515 (3.8%) |  |
| Luxembourg | 670 (1.8%) | 251 (1.9%) |  |
| Slovenia | 1,974 (5.3%) | 819 (6.1%) |  |
| Spain | 3,352 (9.1%) | 821 (6.1%) |  |
| Sweden | 2,612 (7.1%) | 466 (3.5%) |  |
| Switzerland | 2,354 (6.4%) | 269 (2%) |  |
| NA | 0 (0%) | 5,706 (42.3%) |  |

Note. SD: standard deviation, NA: missing information. N (included) + N (excluded) = 50,392 = Participants who provided 3 consecutive main interviews across waves 4, 5, 6, 7, and 8 at least once.

P-values of univariate associations with multimorbidity were computed using two-sided T-tests and chi-square tests (*χ2*) for continuous and categorical variables, respectively.

^a^ One observation corresponds to an observation across 3 consecutive waves.

**Figure S1.** Illustration of the theoretical model, depicting A) overall association B) mediation pathways and C) moderated mediation. Note: Moderators = age, gender, educational attainment, household financial strain. *a_i_b_i_* = indirect effect through respective mediator, *c’* = direct effect, *c* = *c’* + *a_i_b_i_*, = total effect, *w* = wave of measurement


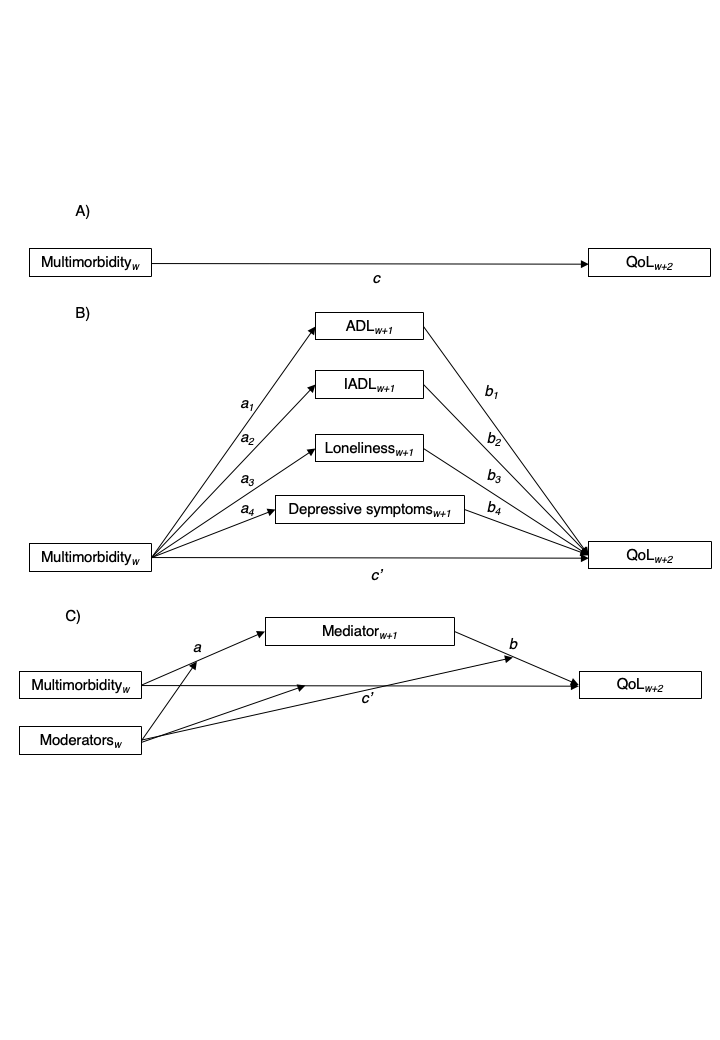


**Figure S2.** Longitudinal mediation model over the five survey waves. Mediator = ADL, IADL, loneliness, depressive symptoms. W4-8 = waves of data collection (approx. 2 years apart). QoL = Quality of Life.

**
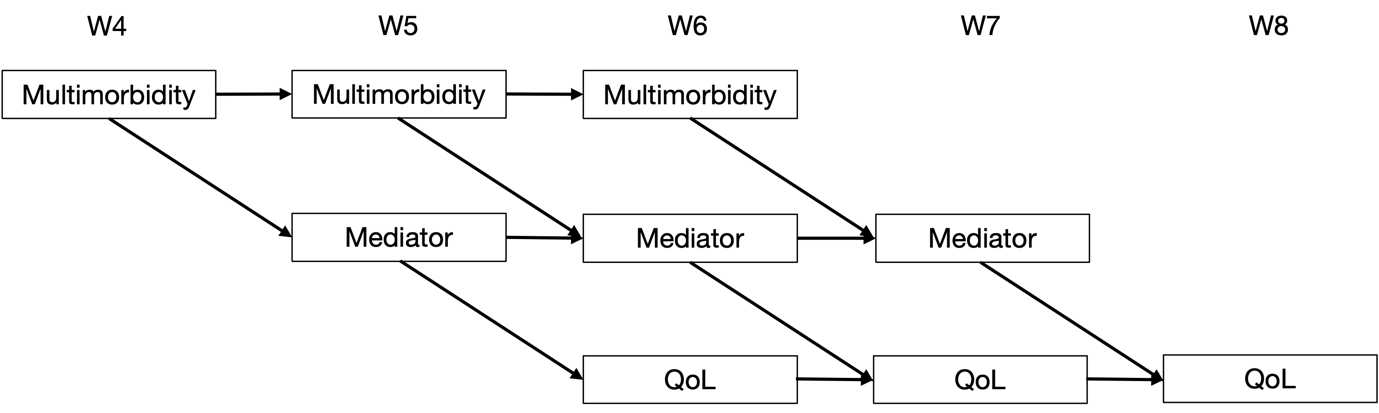
**

**Figure S3.** Flow chart of participant inclusion. Note that one observation (Obs) here means an observation across three consecutive waves.


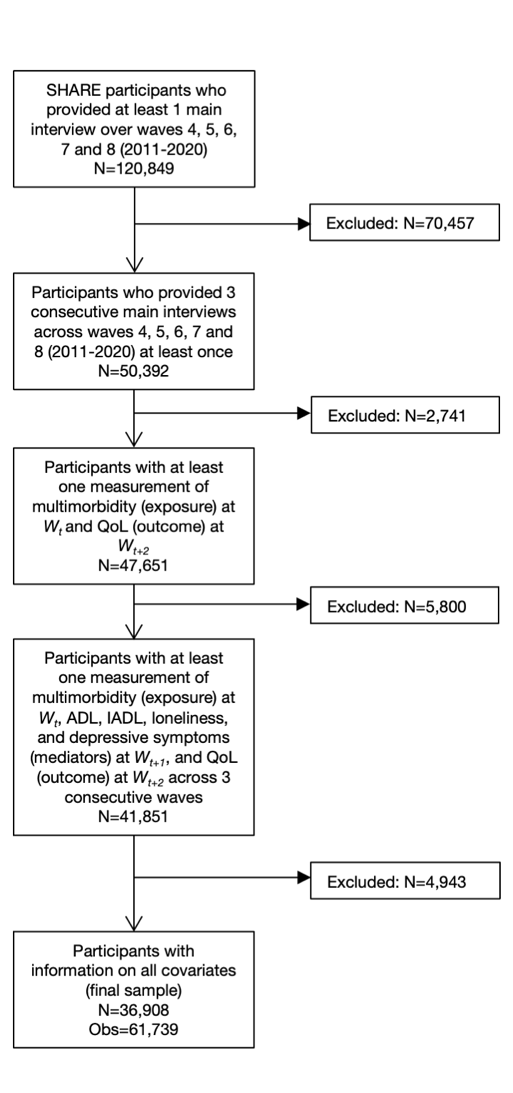

Supplement: igad047_suppl_Supplementary_Material [file igad047_suppl_supplementary_material.docx]
